# Supplementary material for: The cryo-EM structure of a γ-TuSC elucidates architecture and regulation of minimal microtubule nucleation systems
Source: Nat Commun. 2020 Nov 11;11:5705. doi: 10.1038/s41467-020-19456-8 (PMC7658347; doi:10.1038/s41467-020-19456-8)
Supplement: Supplementary file 1 — Supplementary Information [file 41467_2020_19456_MOESM1_ESM.pdf]

Supplementary Information

The cryo-EM structure of a γ-tubulin small complex provides insights into the architecture and regulation of a minimal microtubule nucleation system

Erik Zupa[1], Anjun Zheng[1], Annett Neuner[1], Martin Würtz[1], Peng Liu[1], Anna Böhler[1], Elmar Schiebel[1], Stefan Pfeffer[1]

[1] Zentrum für Molekulare Biologie der Universität Heidelberg, DKFZ-ZMBH Allianz, Im Neuenheimer Feld 282, 69120 Heidelberg, Germany

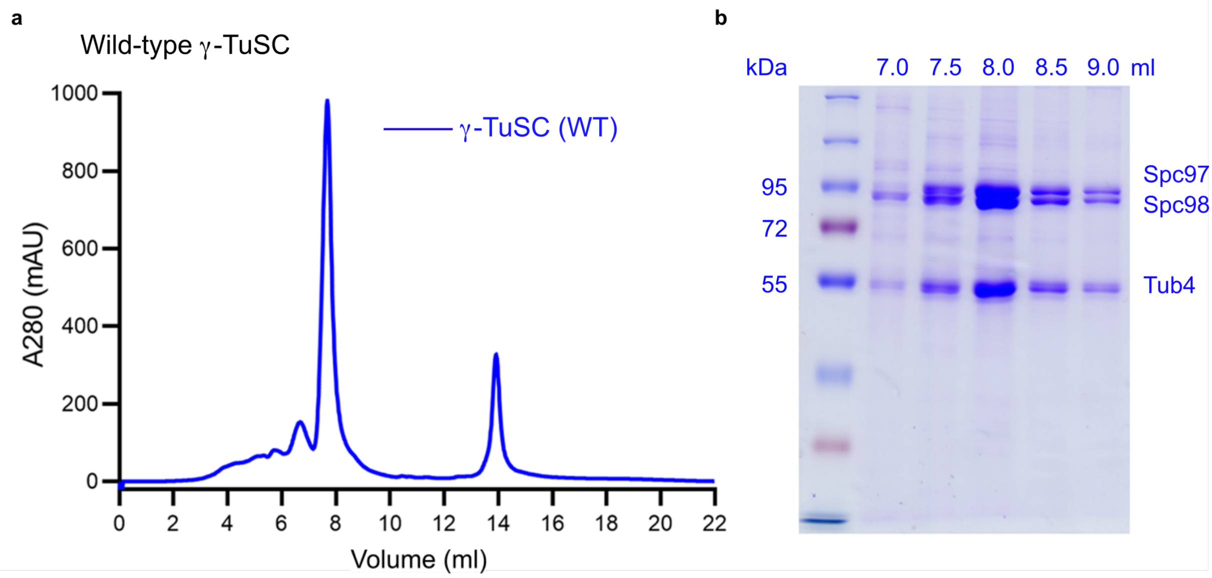

Supplementary Fig. 1. Purification of *C. albicans* wild-type γ-TuSC. a, Anion-exchange chromatography result of wild-type γ-TuSC after Ni-NTA purification. Data points of the absorption curve are included in the Source Data. **b,** SDS-PAGE (CBB) analysis of wild-type γ-TuSC peak fractions. An uncropped raw image of the SDS-PAGE gel is included in the Source Data. The experiment was repeated four times with the same outcome.

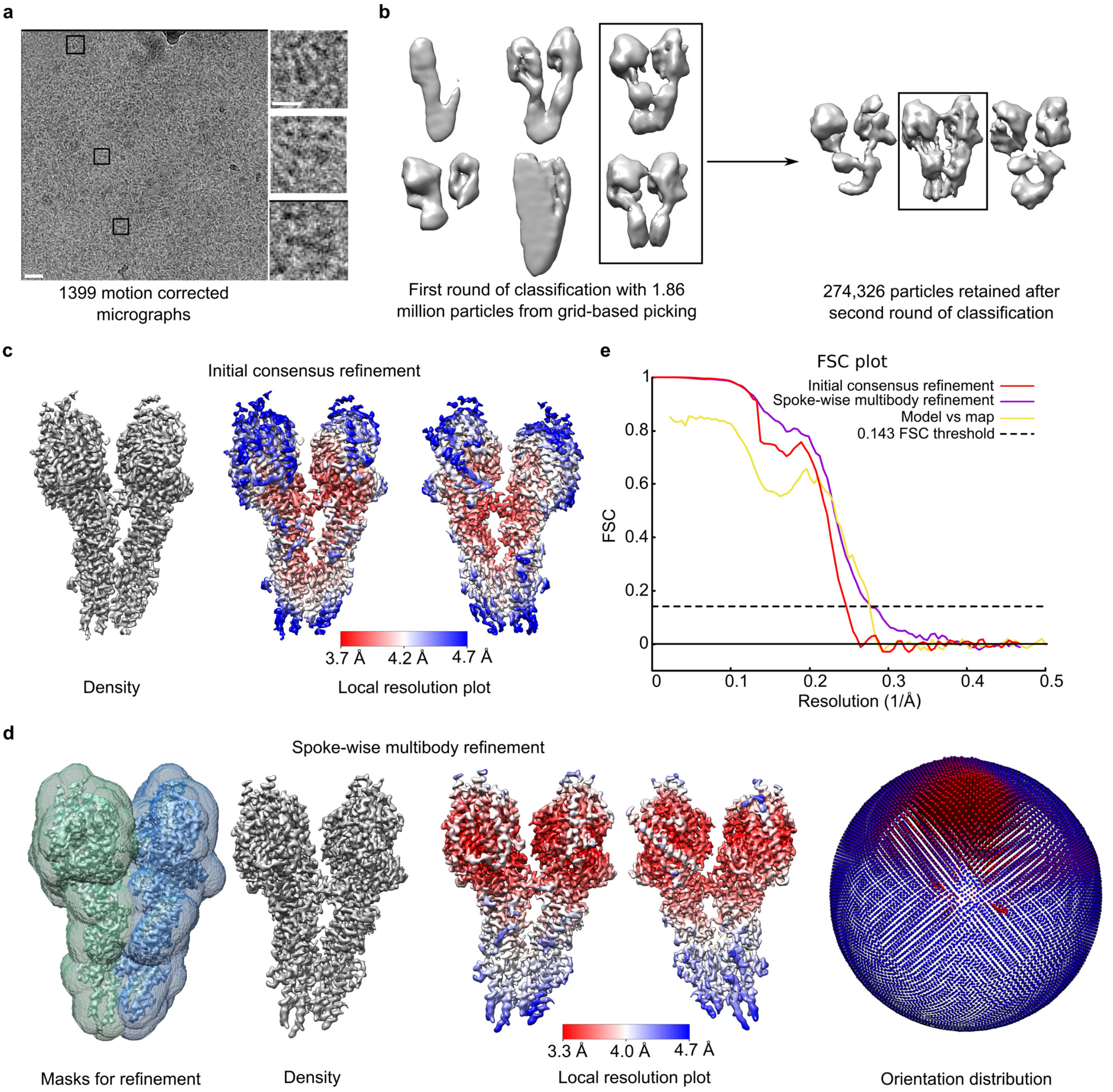

Supplementary Fig. 2. Cryo-EM single particle analysis workflow. a, Representative micrograph of the purified γ-TuSC. 1399 micrographs were acquired in two imaging sessions on two different cryo-EM grids. Three selected particles, aligned on the right side to the micrograph, are shown at higher magnification. Scale bars correspond to 30 nm and 10 nm, respectively. **b,** Particles were extracted from the micrographs based on a regular grid with 10 nm spacing and computationally sorted via several consecutive steps of 3D classification. Due to the large number of particles, the initial round of 3D classification was performed on four subsets in parallel. Classes are shown for one of the subsets. Classes retained for further processing are highlighted. **c**, Initial consensus refinement of the retained particles produced a cryo-EM density (grey) at 4.1 Å global resolution. Local resolution is highest at the interface between the two spokes and ranges from 3.7 to ~ 5 Å. Local resolution is color-coded as indicated. **d**, The γ-TuSC particles were subjected to multibody refinement using the depicted

masks, comprising one spoke each. The output density segments were combined into one composite density at 3.6 Å global resolution. Notably, local resolution increased overall and highest local resolution is achieved in the center of each spoke. Local resolution is color-coded as indicated. Please note that the color-coded range is different from (c). The angular distribution of particle views is shown in the same orientation. **e**, Mask-corrected Fourier shell correlation between the two independently refined half set reconstructions before (red) and after (purple) multibody refinement and Fourier shell correlation between the full reconstruction and the atomic model for the γ-TuSC. The FSC = 0.143 threshold is indicated by a dashed line. Data points for all plotted FSC curves are included in the Source Data.

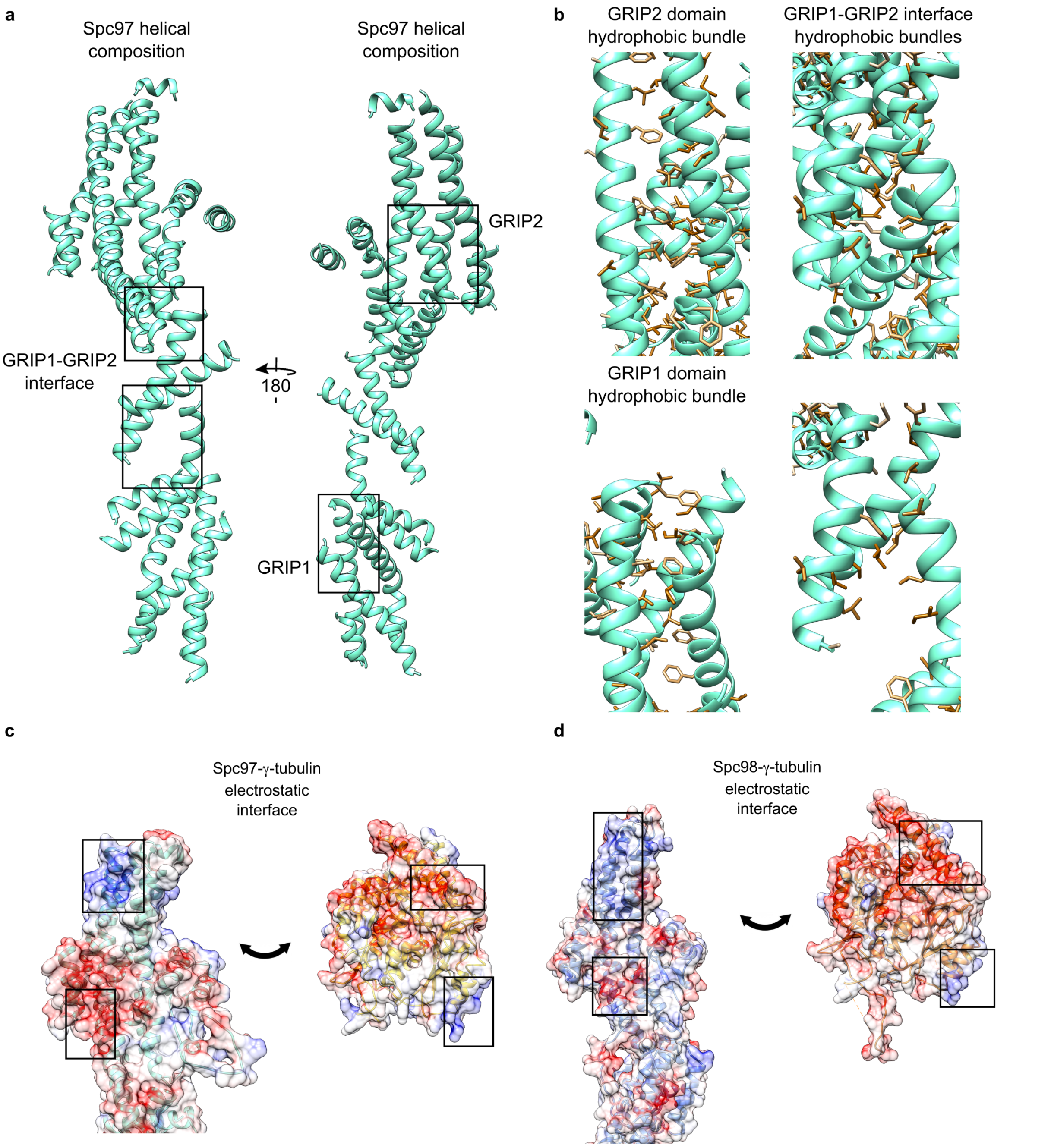

Supplementary Fig. 3. Molecular interactions mediating Spc97/98-γ-tubulin heterodimer formation. a, Overall arrangement of α-helices in Spc proteins. Spc97 is shown as a representative. **b**, The Spc core fold is stabilized by hydrophobic interactions within helical bundles and between the GRIP1 and GRIP2 domains. Hydrophobic amino acid residues are shown in brown. Zoomed views as indicated in (a). **c**, **d** Complementary charged patches on the Spc GRIP2 domain (left) and γ-tubulin (right) mediate electrostatic interactions between γ-tubulin and Spc97 (c) or Spc98 (d), respectively. Surface representation of the atomic model, colored according to charge (red: negative, blue: positive, white: no charge).

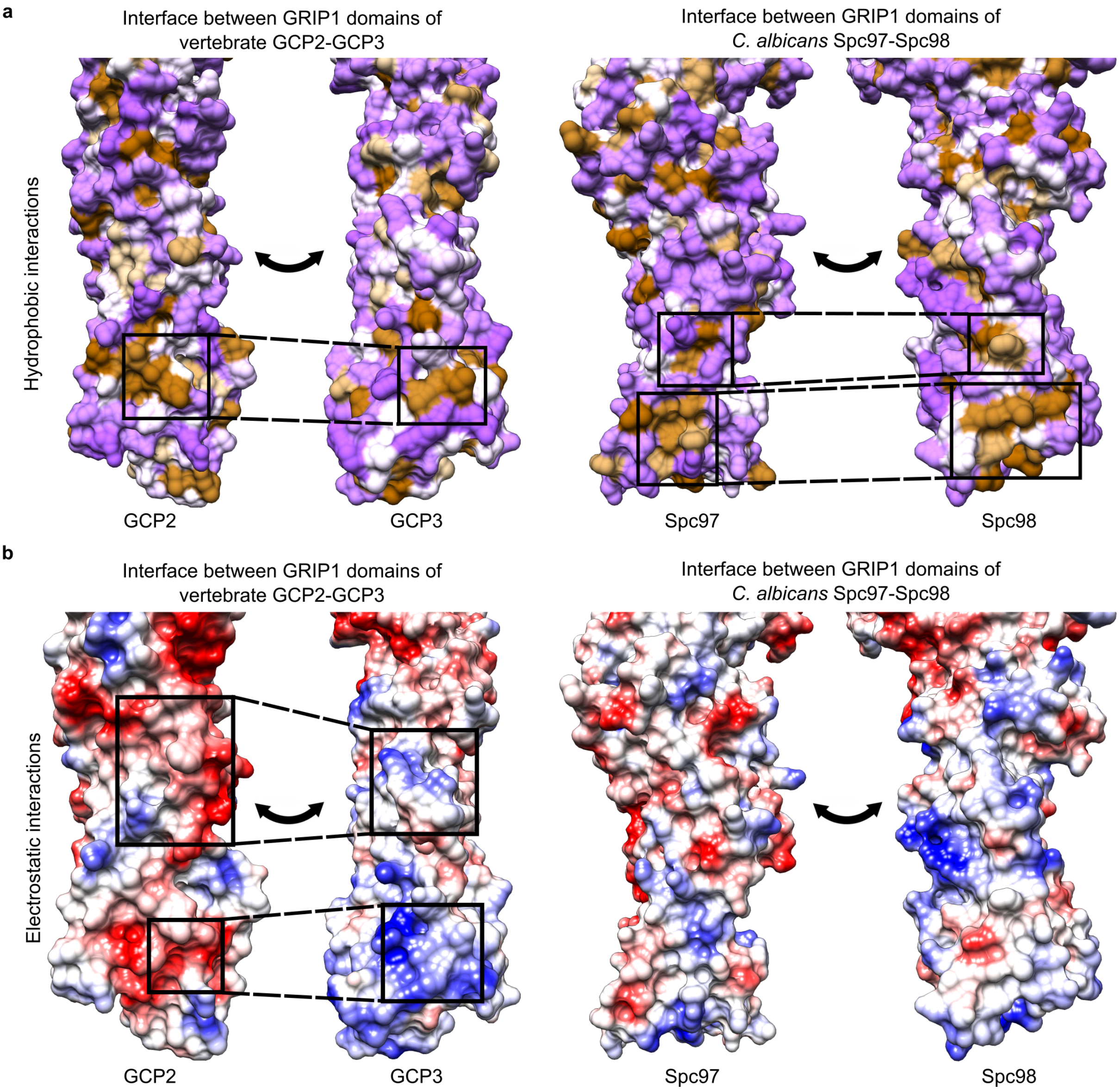

Supplementary Fig. 4. Molecular interactions mediating GRIP1 domain interaction in *C. albicans* and human γ-TuSC units. a, Interface between GRIP1 domains in human GCP2 and GCP3 (left) and *C. albicans* Spc97 and Spc98 (right). The models are shown in surface representation and color-coded according to hydrophobicity (brown: hydrophobic; purple: hydrophilic). Interacting hydrophobic patches are indicated. **b**, Interface between GRIP1 domains in human GCP2 and GCP3 (left) and *C. albicans* Spc97 and Spc98 (right). The models are shown in surface representation and color-coded according to charge (red: negative, blue: positive, white: no charge). Complementary charged patches on the two surfaces are indicated.

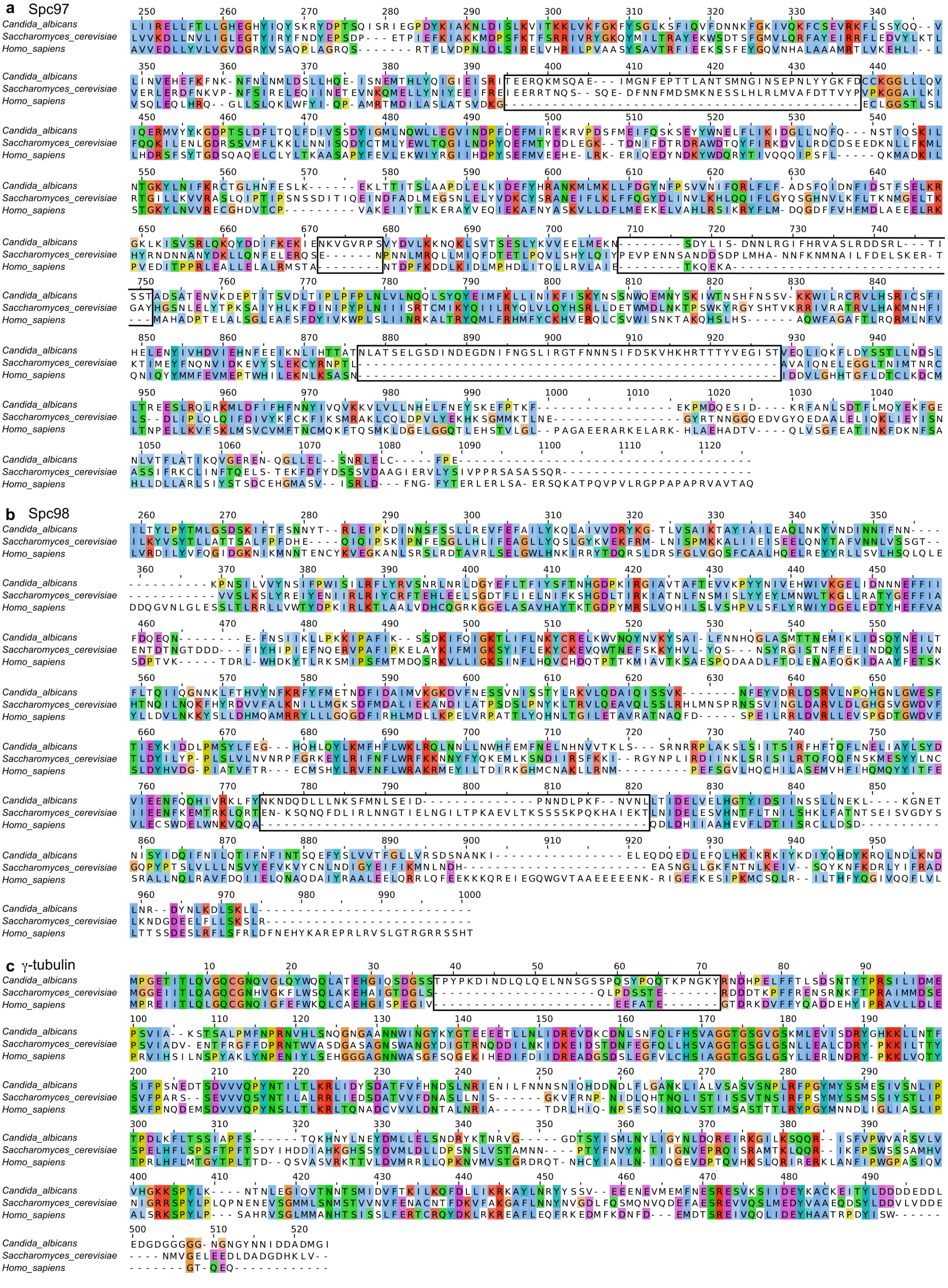

Supplementary Fig. 5. Sequence alignment of *C. albicans*, *S. cerevisiae* and human γ-TuSC subunits. Sequences of Spc97 **(a)**, Spc98 **(b)** and γ-tubulin **(c)** are shown. Fungi-specific insertions that were modelled (completely or partially) are highlighted by black boxes. The

same insertions have been mapped back to the structure in Supplementary Fig. 6. Numbering is according to the consensus sequence alignment.

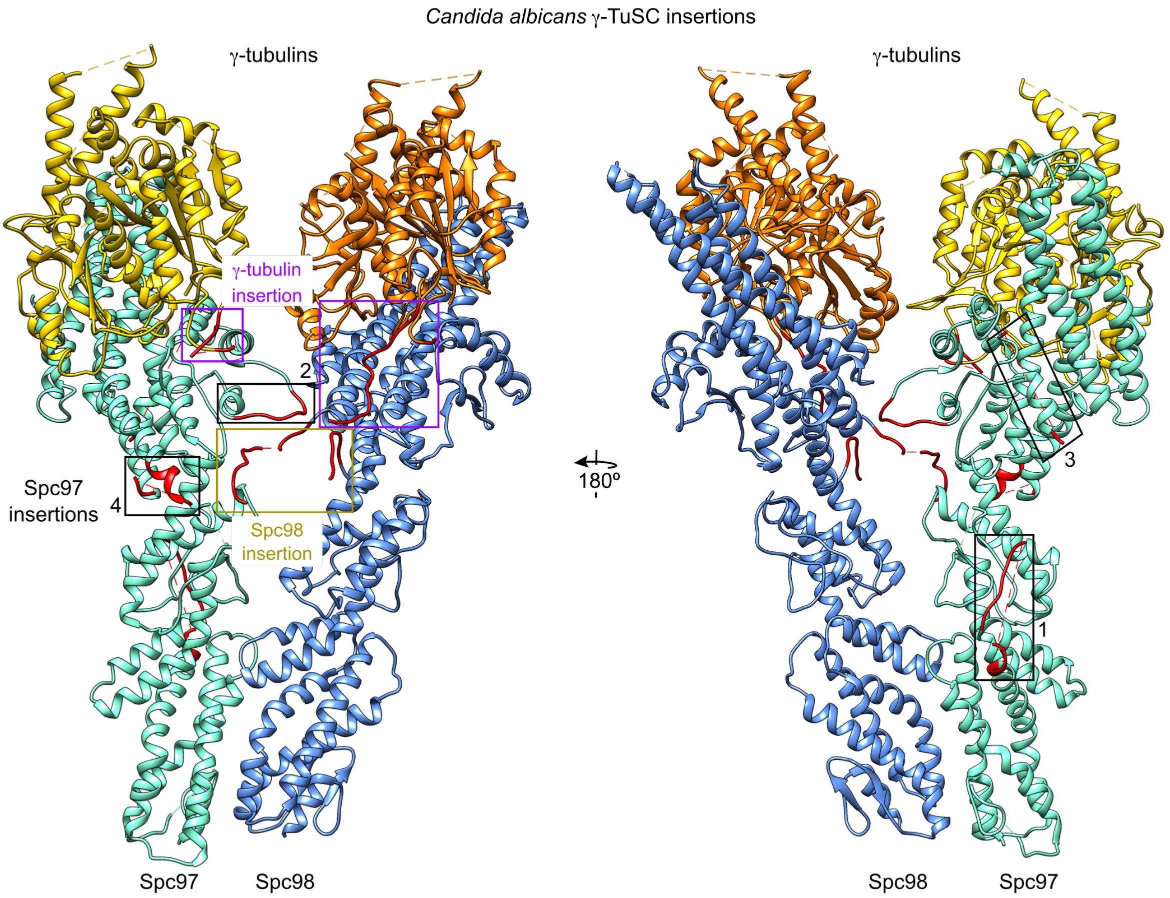

Supplementary Fig. 6. Location of modelled fungi-specific insertions in γ-TuSC subunits. Modelled fungi-specific insertions (red) of Spc97 (black boxes and numbers), Spc98 (green box) and γ-tubulin (purple boxes) are shown in context of the full model. Insertions in Spc97 are numbered from N- to C-terminus. The corresponding sequence alignments are shown in Supplementary Fig. 5. Coloring as in Fig. 1a.

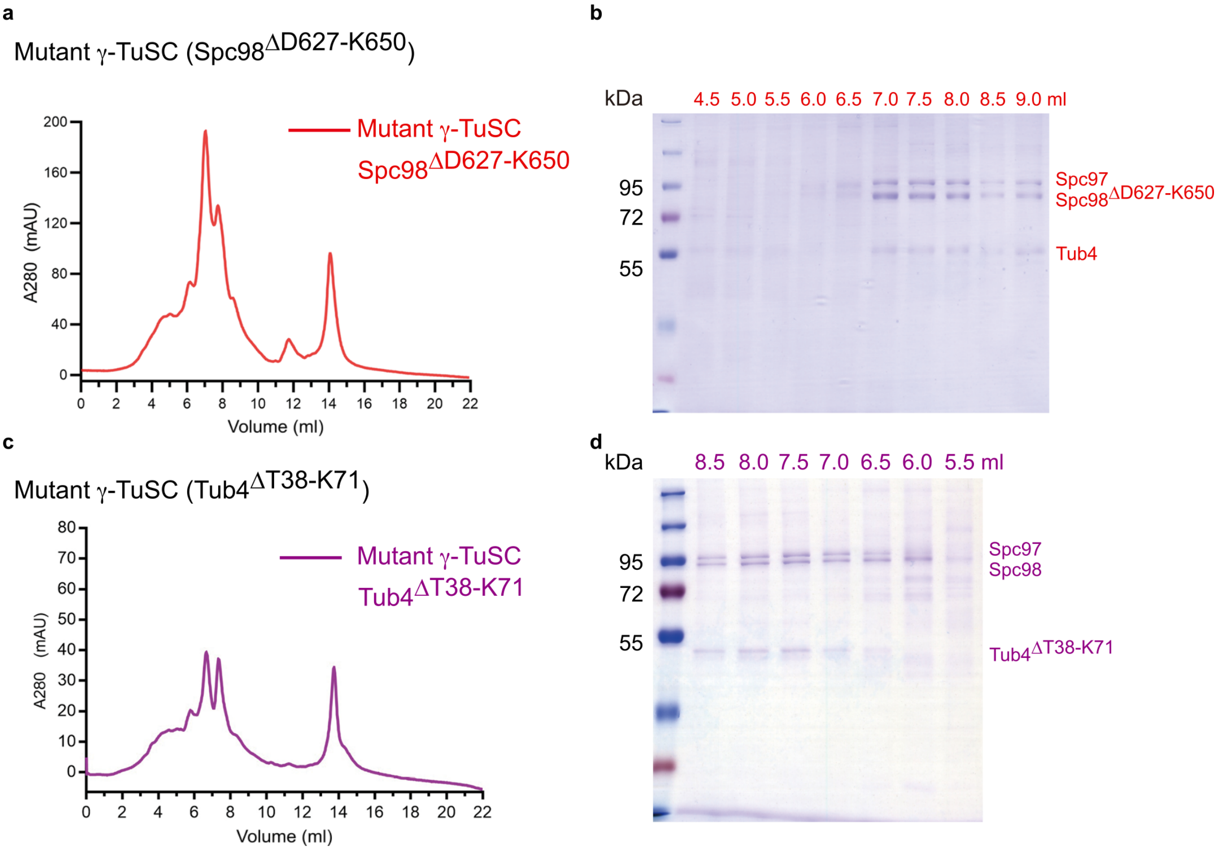

Supplementary Fig. 7. Purification of mutant *C. albicans* γ-TuSC with deleted insertions in either Spc98 or Tub4. a, Anion-exchange chromatography result of the Spc98 mutant of γ-TuSC (Spc98$^{\Delta D627\text{-}K650}$) after Ni-NTA purification. Data points of the absorption curve are included in the Source Data. **b,** SDS-PAGE (CBB) analysis of peak fractions from (a). An uncropped raw image of the SDS-PAGE gel is included in the Source Data. The experiment was repeated two times with the same outcome. **c,** Anion-exchange chromatography result of the Tub4 mutant of γ-TuSC (Tub4$^{\Delta T38\text{-}K71}$) after Ni-NTA purification. Data points of the absorption curve are included in the Source Data. **d,** SDS-PAGE (CBB) analysis of the peak fractions from (c). An uncropped raw image of the SDS-PAGE gel is included in the Source Data. The experiment was repeated two times with the same outcome.

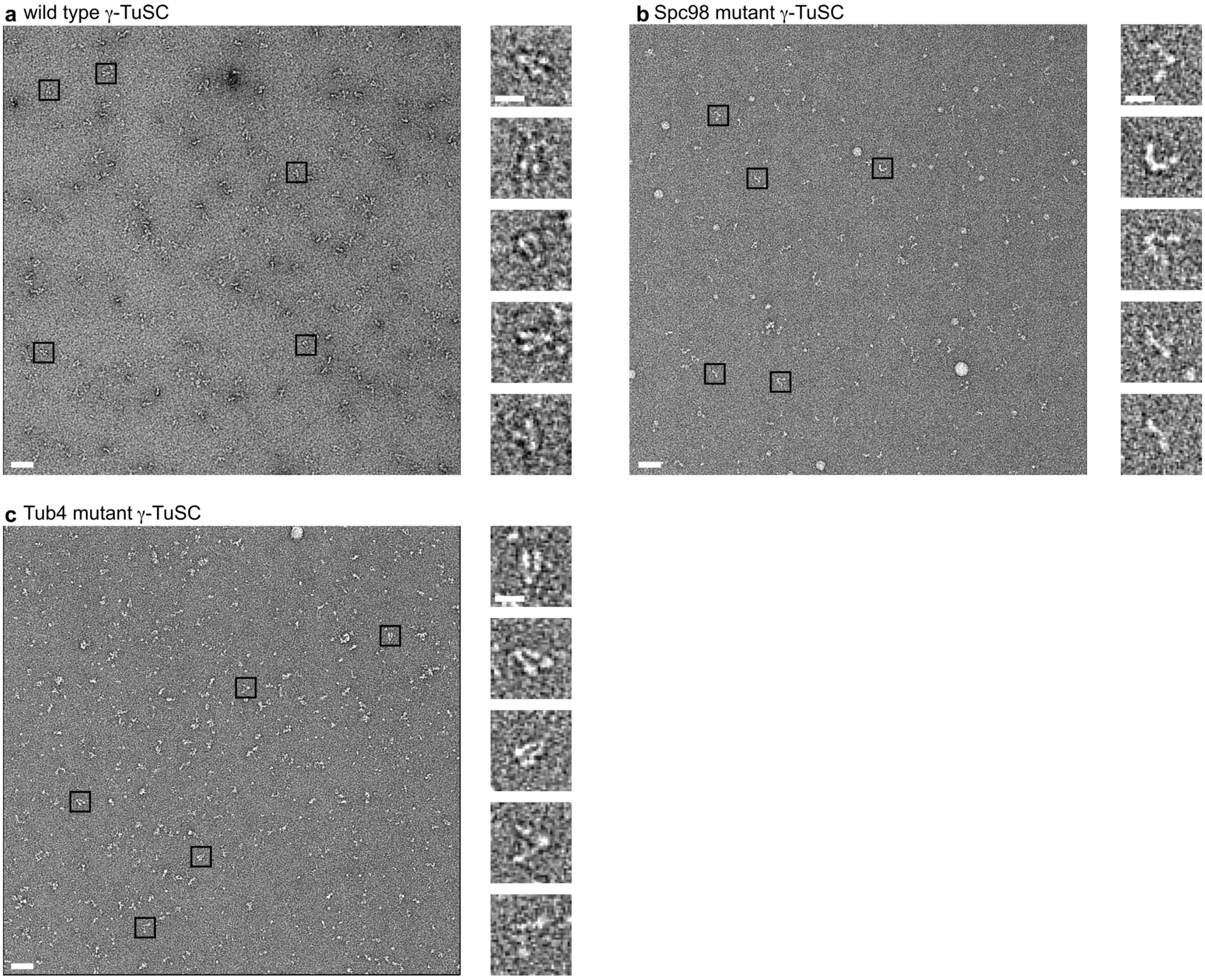

Supplementary Fig. 8. Negative stain EM analysis of wild-type and mutant γ-TuSC. One representative micrograph is shown together with five selected particles at higher magnification for the wild-type γ-TuSC (**a**), the Spc98 mutant (Spc98$^{\Delta D627\text{-}K650}$) γ-TuSC (**b**) and the Tub4 mutant (Tub4$^{\Delta T38\text{-}K71}$) γ-TuSC (**c**). Scale bars correspond to 50 nm and 20 nm for the micrographs and particle images, respectively. Data for each γ-TuSC construct were acquired in one imaging session. The numbers of micrographs used were 997 for the wild-type γ-TuSC, 821 for the Spc98$^{\Delta D627\text{-}K650}$ mutant γ-TuSC and 1147 for the Tub4$^{\Delta T38\text{-}K71}$ mutant γ-TuSC.

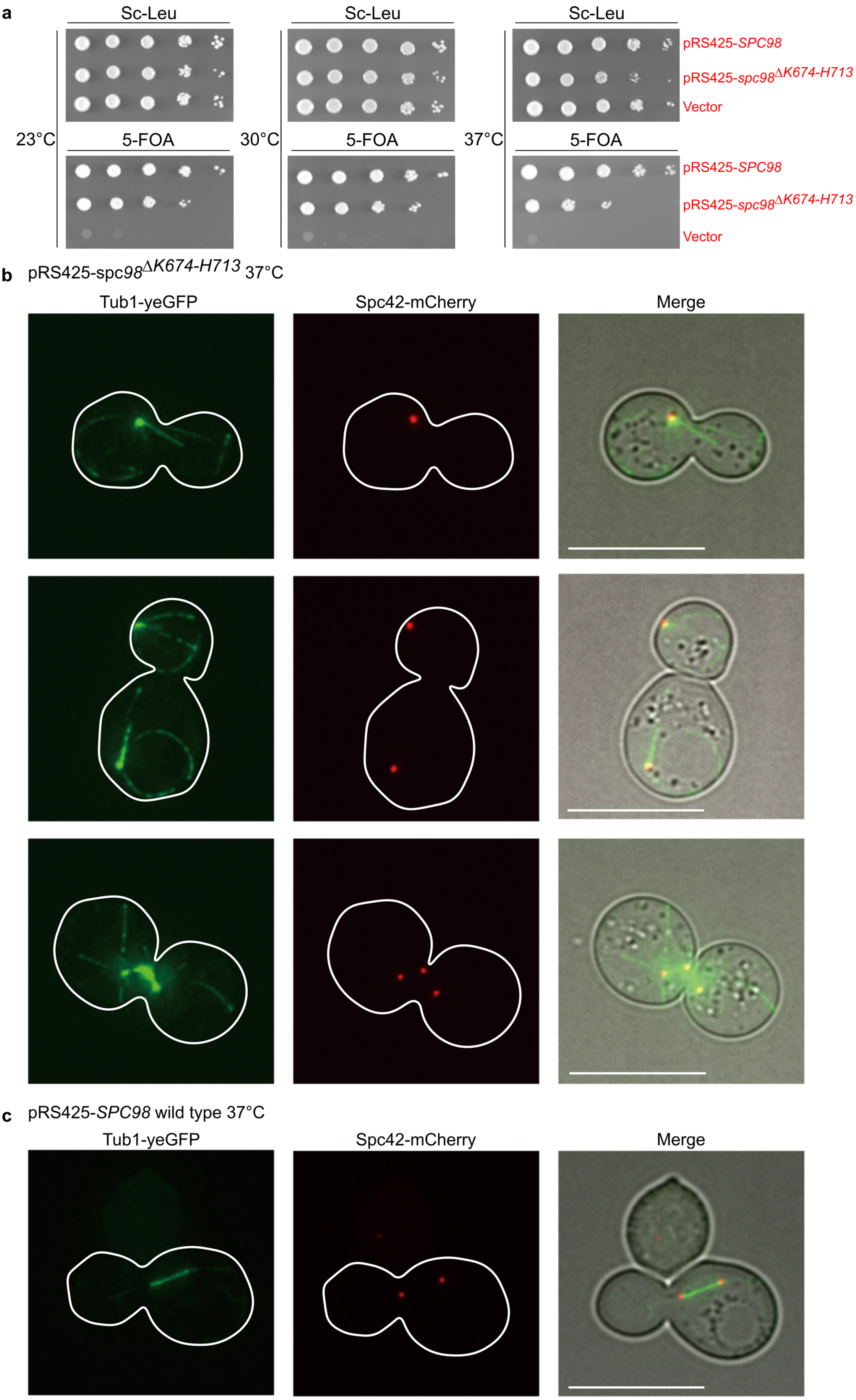

a
Sc-Leu
5-FOA
23°C
30°C
37°C
pRS425-SPC98
pRS425-spc98ΔK674-H713
Vector
b pRS425-spc98ΔK674-H713 37°C
Tub1-yeGFP
Spc42-mCherry
Merge
c pRS425-SPC98 wild type 37°C
Tub1-yeGFP
Spc42-mCherry
Merge

Supplementary Fig. 9. *S. cerevisiae* mutant Spc98$^{\Delta K674\text{-}H713}$ causes defects in mitosis. a, Strain ESM243-2 (*MATa ura3-52 lys2-801 ade2-101::pRS402-GFP-TUB1 trp1Δ63 his3Δ200 leu2Δ1 Δspc98::HIS3 pRS316-SPC98 SPC42-mCherry-natNT2*) was transformed with pRS425-*SPC98*, pRS425-*spc98*$^{\Delta K674\text{-}H713}$ and pRS425 (vector). Transformants were tested for growth after serial dilution at the indicated temperatures on SC-Leu and 5-FOA plates for 2 - 3 days. The growth behavior is identical to strain ESM243-1 shown in Fig. 3d. **b-c,** Cells of *spc98*$^{\Delta K674\text{-}H713}$ (**b**) or *SPC98* (**c**) expressing *TUB1-yeGFP* (green) and *SPC42-mCherry* (red) were cultured at 37℃ for 3 hours. The Tub1-yeGFP (green) and Spc42-mCherry (red) fluorescent signals and the merged signals with phase contrast are shown. Scale bars, 10 µm. The experiments were repeated two times with the same outcome.

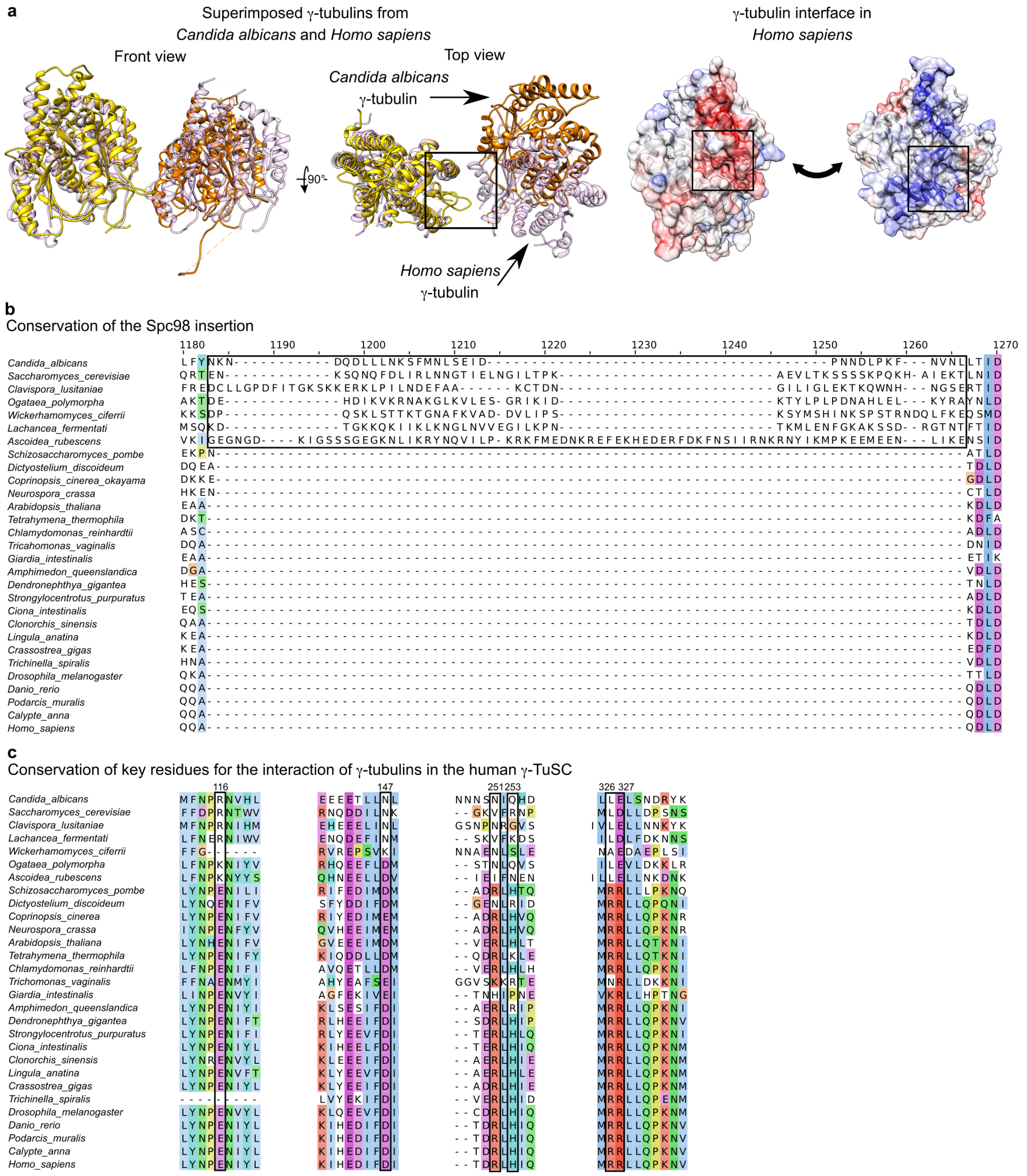

Supplementary Fig. 10. The fungi-specific Spc98 insertion may replace direct interaction of neighboring γ-tubulins during evolution. a, Left: Superposition of atomic models for the two γ-tubulin molecules as arranged in the *C. albicans* (yellow, orange) and human (pink) γ-TuSC units. The γ-tubulin interaction area in the human γ-TuSC is indicated. Right: Complementary charged patches on the two γ-tubulin molecules mediate electrostatic interactions in the human γ-TuSC. Surface representation of the atomic model, colored according to charge (red: negative, blue: positive, white: no charge). **b,** Spc98 sequences were aligned for a broad range of organisms and the region of the fungi-specific Spc98 insertion is shown. The full multiple sequence alignment is included in the Source Data. Numbering is

according to the consensus sequence alignment. **c,** γ-tubulin sequences were aligned for the organisms in (b) and the regions including key residues (boxed) for the electrostatic interaction of γ-tubulins in the human γ-TuSC are shown. The full multiple sequence alignment is included in the Source Data.

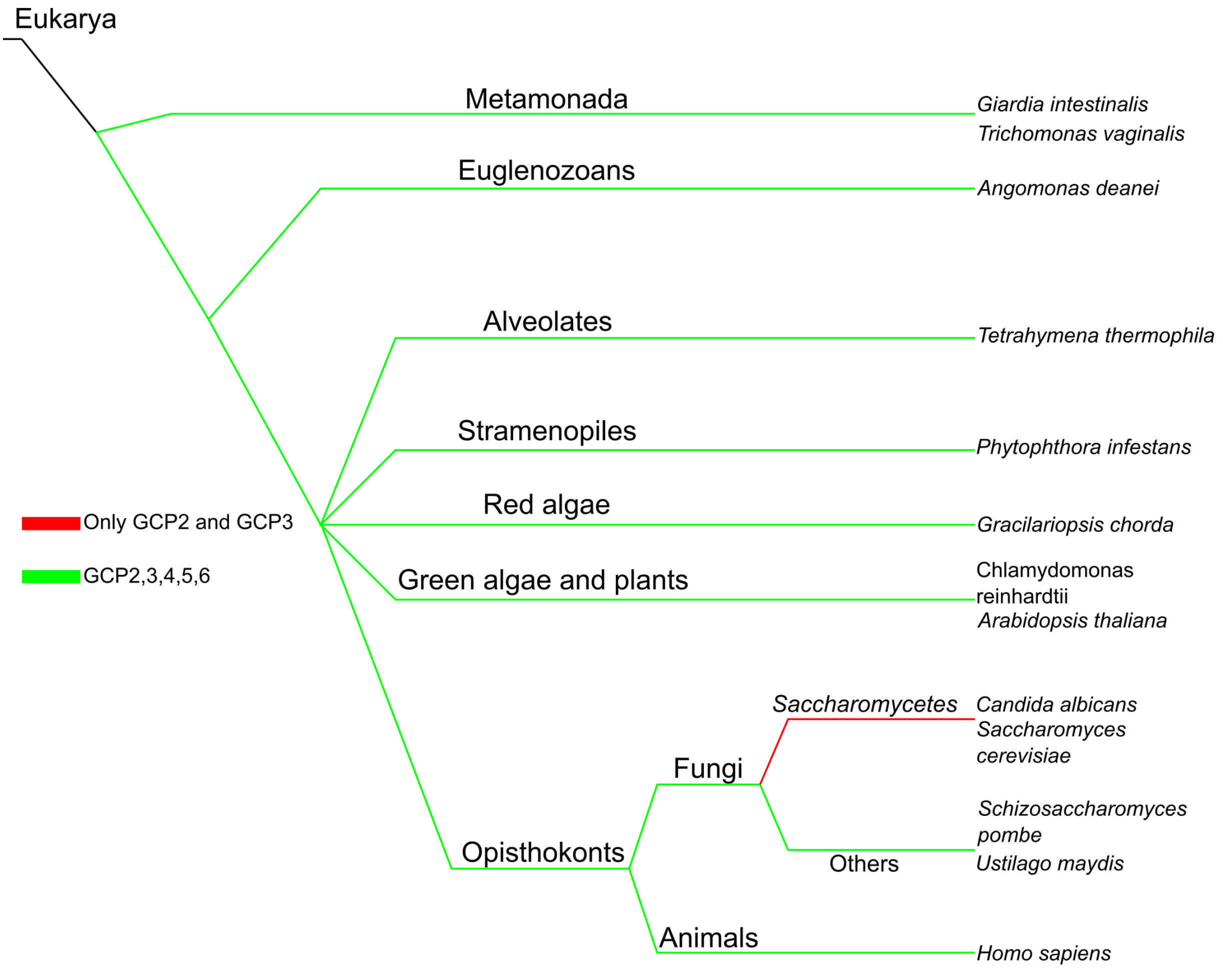

Supplementary Fig. 11. The γ-TuSC system in *Saccharomycetes* emerged from the γ-TuRC system by compositional simplification. Phylogenetic tree of life for the major branches of eukaryotes based on genomic similarities. Representative organisms of green branches possess homologs for all five GCP proteins known to be required for formation of the γ-TuRC. *Saccharomycetes* (red branch) possess only homologs for GCP2/Spc97 and GCP3/Spc98. Sequence identifiers for the GCP homologs of all analyzed organisms are included in Supplementary Tables 1 and 2.

Supplementary Table 1: Sequence identifiers of GCP subunits identified in various eukaryotes

Organism	Phylum	GCP2	GCP3	GCP4	GCP5	GCP6
Giardia Intestinalis	*Metamonada*	A8BD62	A8BFK8	KAE8304190.1	ESU36978.1	A8BUR1
Trichomonas vaginalis	*Metamonada*	A2E313	A2E3S1	XP_001325680.1	XP_001323803.1	>XP_001328726.1
Angomonas deanei	*Euglenozoa*	EPY32211.1	EPY37939.1	EPY31448.1	EPY34001.1	EPY30063.1
Tetrahymena thermophila	*Alveolates*	Q23AE3	Q22ZA9	XP_001025759.3	XP_001014477.2	XP_001015474.3
Phytophthora infestans	*Stramenopiles*	KAF4031510.1	KAF4046598.1	KAF4031595.1	KAF4137761.1	KAF4030965.1
Gracilariopsis chorda	*Rhodophyta*	PXF41098.1	PXF45651.1	PXF41306.1	-	PXF43115.1
Chlamydomonas reinhardtii	*Chlorophyta*	A8J5J8	A8JBY6	XP_001689495.1	XP_001699475.1	PNW77310.1
Arabidopsis thaliana	*Spermatophyta*	Q9C5H9	Q9FG37	NP_190944.2	NP_565235.3	NP_189947.2
Candida albicans	*Ascomycota*	Q59PZ2	A0A1D8PS42	-	-	-
Homo sapiens	*Chordata*	Q9BSJ2	Q96CW5	Q9UGJ1	Q96RT8	Q96RT7

Supplementary Table 2: Sequence identifiers of GCP subunits identified in various fungi

Organism	Class	GCP2	GCP3	GCP4	GCP5	GCP6
Batrachochytrium dendrobatidis	*Chytridiomycetes*	XP_006676384.1	OAJ42679.1	OAJ44291.1	OAJ37135.1	XP_006681750.1
Ustilago maydis	*Ustilaginomycetes*	XP_011388094.1	XP_011386949.1	XP_011388935.1	XP_011386225.1	XP_011387192.1
Pneumocystis jirovecii	*Pneumocystis*	CCJ30183.1	XP_018229083.1	XP_018230983.1	XP_018228508.1	CCJ29560.1
Schizosaccharomyces pombe	*Schizosaccharomycetales*	Q9Y705	Q9USQ2	Q9P7R5	Q9UT52	P87244
Candida albicans	*Saccharomycetes*	Q59PZ2	A0A1D8PS42	-	-	-
Saccharomyces cerevisiae	*Saccharomycetes*	P38863	P53540	-	-	-
Clavispora lusitaniae	*Saccharomycetes*	XP_002617032.1	OVF11036.1	-	-	-
Ogataea polymorpha	*Saccharomycetes*	XP_018211750.1	XP_018213099.1	-	-	-
Wickerhamomyces ciferrii	*Saccharomycetes*	XP_011273399.1	XP_011277192.1	-	-	-
Lachancea fermentati]	*Saccharomycetes*	SCW03932.1	SCW02410.1	-	-	-
Ascoidea rubescens	*Saccharomycetes*	XP_020046155.1	XP_020044979.1	-	-	-

Supplementary Table 3: List of primers

Construct	Primer name	Primer sequence
C. albicans tub4$^{\Delta T38-K71}$	cTub4-pFB-F0	gtttcggtctccacgcatcg
C. albicans tub4$^{\Delta T38-K71}$	cTub4-pFB-R	tattccggattattcataccgtcccac
C. albicans tub4$^{\Delta T38-K71}$	cTub4-pFB-R	ctttgaattccgcgcgcttcg
C. albicans tub4$^{\Delta T38-K71}$	cTub4-T38-K71-F	gtccgacgggagctcatatagaaatgatcatc
C. albicans tub4$^{\Delta T38-K71}$	cTub4-T38-K71-R	gatgatcatttctatatgagctcccgtcggac
C. albicans spc98$^{\Delta D627-K650}$	cSpc98-pFBHTA-F	gattacgatatcccaacgac
C. albicans spc98$^{\Delta D627-K650}$	cSpc98-pFBHTA-R	gtaggcctttgaattccggatc
C. albicans spc98$^{\Delta D627-K650}$	cSpc98-D627-K650-F	gcttttttacaataaaaattttaatgttaatctgttg
C. albicans spc98$^{\Delta D627-K650}$	cSpc98-D627-K650-R	caacagattaacattaaaatttttattgtaaaaaagc
S. cerevisiae spc98$^{\Delta K674-H713}$	Spc98-D674-H713-F	cgccagggttttcccagtc
S. cerevisiae spc98$^{\Delta K674-H713}$	Spc98-D674-H713-F1	ctgcaacgcacagagaatgcaatcgaaaagacgctg
S. cerevisiae spc98$^{\Delta K674-H713}$	Spc98-D674-H713-R1	cagcgtcttttcgattgcattctctgtgcgttgcag
S. cerevisiae spc98$^{\Delta K674-H713}$	Spc98-D674-H713-R	caggaaacagctatgac
