# Supplementary material for: The cryo-EM structure of a γ-TuSC elucidates architecture and regulation of minimal microtubule nucleation systems
Source: Nat Commun. 2020 Nov 11;11:5705. doi: 10.1038/s41467-020-19456-8 (PMC7658347; doi:10.1038/s41467-020-19456-8)
Supplement: Supplementary file 3 — Reporting Summary [file 41467_2020_19456_MOESM3_ESM.pdf]

nature research

Corresponding author(s): Stefan Pfeffer, Elmar Schiebel

Last updated by author(s): Oct 14, 2020

Reporting Summary

Nature Research wishes to improve the reproducibility of the work that we publish. This form provides structure for consistency and transparency in reporting. For further information on Nature Research policies, see our Editorial Policies and the Editorial Policy Checklist.

Statistics

For all statistical analyses, confirm that the following items are present in the figure legend, table legend, main text, or Methods section.

n/a	Confirmed	
☐	☒	The exact sample size (*n*) for each experimental group/condition, given as a discrete number and unit of measurement
☐	☒	A statement on whether measurements were taken from distinct samples or whether the same sample was measured repeatedly
☒	☐	The statistical test(s) used AND whether they are one- or two-sided<br>*Only common tests should be described solely by name; describe more complex techniques in the Methods section.*
☒	☐	A description of all covariates tested
☒	☐	A description of any assumptions or corrections, such as tests of normality and adjustment for multiple comparisons
☒	☐	A full description of the statistical parameters including central tendency (e.g. means) or other basic estimates (e.g. regression coefficient) AND variation (e.g. standard deviation) or associated estimates of uncertainty (e.g. confidence intervals)
☒	☐	For null hypothesis testing, the test statistic (e.g. *F*, *t*, *r*) with confidence intervals, effect sizes, degrees of freedom and *P* value noted<br>*Give P values as exact values whenever suitable.*
☒	☐	For Bayesian analysis, information on the choice of priors and Markov chain Monte Carlo settings
☒	☐	For hierarchical and complex designs, identification of the appropriate level for tests and full reporting of outcomes
☒	☐	Estimates of effect sizes (e.g. Cohen's *d*, Pearson's *r*), indicating how they were calculated

Our web collection on statistics for biologists contains articles on many of the points above.

Software and code

Policy information about availability of computer code

Data collection	Datasets for cryo-EM were collected with SerialEM (3.7) at a Titan Krios TEM (Thermofisher Scientific) equipped with a K2 direct electron detector (Gatan). Datasets for negative stain EM were collected at a Talos L120C with a Ceta 16M camera (Thermofisher Scientific) using EPU 2.4.0.4 (Thermofisher Scientific). Different sequences of γ-TuSC components were obtained from the National Center for Biotechnology Information using the Basic Local Alignment Search Tool (BLAST 2.10.0). Drop out images were acquired on a LAS-4000 machine (Fujifilm Life Science). Images for phenotype analysis of S. cerevisiae Spc98ΔK674-H713 were acquired via the DeltaVision RT system (Applied Precision) with an Olympus IX71 microscope equipped with 100X NA UPlanSAPO objective lense (Olympus) using SoftWoRx 7.2.0 software (Applied Precision).
Data analysis	Cryo-EM data were processed using Relion 3.0-Beta, MotionCorr 2.0 and gCTF 1.06. All density map-related figures were prepared in Chimera 1.13.1 and ChimeraX 0.9. Atomic modelling was performed in Coot 0.9. Refinement and final flexible fitting of the model was performed using the website tool NAMDinator (https://namdinator.au.dk, accessed in April 2020), and Phenix 1.14. Analysis and vector visualisation of γ-TuSC conformations was performed in PyMol 2.1. Negative stain data were processed using Relion 3.0-Beta and gCTF 1.06. Alignment of Spc97 and Spc98 sequences was performed in PROMALS-3D online tool (http://prodata.swmed.edu/promals3d/promals3d.php, accessed on May 2020) and for γ-tubulin using the MAFFT online tool version 7. Aligned sequences were displayed and analysed in Jalview (2101-VM). Residues forming the interfaces in the γ-TuSC were identified using PDBePISA 1.48. Image processing and analysis was performed in a semi-automated manner with the open-source ImageJ 1.46r software package (National Institutes of Health). Anion-exchange chromatography data were plotted with Prism 6.1 (GraphPad Software).

For manuscripts utilizing custom algorithms or software that are central to the research but not yet described in published literature, software must be made available to editors and reviewers. We strongly encourage code deposition in a community repository (e.g. GitHub). See the Nature Research guidelines for submitting code & software for further information.

Data

Policy information about availability of data

All manuscripts must include a data availability statement. This statement should provide the following information, where applicable:

- Accession codes, unique identifiers, or web links for publicly available datasets
- A list of figures that have associated raw data
- A description of any restrictions on data availability

The Cryo-EM density of the γ-TuSC filtered according to local resolution was deposited in the Electron Microscopy Data Bank (EMDB) under accession code EMD-11835 [https://www.ebi.ac.uk/pdbe/entry/emdb/EMD-11835]. Atomic coordinates for the γ-TuSC were deposited at the Protein Data Bank (PDB) under accession code PDB-7ANZ [https://www.rcsb.org/structure/7ANZ]. Source data associated with Supplementary Figs. 1, 2, 7 and 10 are provided with the paper. The raw cryo-EM micrograph movie stacks are available from the corresponding authors upon request. Published structural data used in this article: PDB-4FFB [https://www.rcsb.org/structure/4FFB], PDB-5W3F [https://www.rcsb.org/structure/5W3F], PDB-1Z5W [https://www.rcsb.org/structure/1Z5W], PDB-3RIP [https://www.rcsb.org/structure/3RIP], PDB-6V6B [https://www.rcsb.org/structure/6V6B], PDB-5FM1 [https://www.rcsb.org/structure/5FM1], PDB-5FLZ [https://www.rcsb.org/structure/5FLZ], EMD-2799 [https://www.ebi.ac.uk/pdbe/entry/emdb/EMD-2799].

Field-specific reporting

Please select the one below that is the best fit for your research. If you are not sure, read the appropriate sections before making your selection.

☒ Life sciences ☐ Behavioural & social sciences ☐ Ecological, evolutionary & environmental sciences

For a reference copy of the document with all sections, see nature.com/documents/nr-reporting-summary-flat.pdf

Life sciences study design

All studies must disclose on these points even when the disclosure is negative.

Sample size	No statistical methods were used to predetermine sample size.<br><br>Cryo-EM data were collected in a sufficient amount for high-resolution reconstruction. Negative stain EM data were collected in a sufficient amount for extensive particle classification and averaging.<br><br>Plasmid shuffle experiment data were obtained in a sufficient amount to characterize growth differences between the strains analyzed. Fluorescence microscopy data for phenotype analysis were acquired in a sufficient amount to characterize differences in cellular microtubule organization between the strains analyzed.
Data exclusions	No data were excluded from analyses.
Replication	Cryo-EM data were acquired in two imaging sessions from two different EM grids with the same outcome. Negative stain EM data was acquired in one imaging session for each of the complexes. For cryo-EM and negative stain EM analyses, the final densities are the averages of thousands of particles.<br><br>The yeast shuffle experiment was repeated 3 times with the same outcome. Purification and SDS-PAGE analysis of the wild-type complex, the Spc98 mutant complex and the gamma-tubulin mutant complex were repeated 4 times, 2 times and 2 times, respectively, always with the same outcome. Fluorescence microscopy experiments were repeated two times with the same outcome.
Randomization	Positions for image acquisition in cryo-EM and Negative stain EM were selected based on particle density. The particles were selected by a computer program (RELION 3.0 Beta) in an unsupervised manner. Reconstructions used two randomized half-sets to prevent over-refinement of the model, and to assess the resolution of the final model.<br><br>Images of the plasmid shuffle experiment were acquired on all plates and no data was excluded, so randomization does not apply. Fluorescense microscopy data were acquired randomly.
Blinding	Analyses of cryo-EM and negative stain EM were not blinded, as they were performed computationally in an almost unsupervised manner as typical in EM analyses. The plasmid shuffle experiment data were not blinded because no data was excluded and no further image analysis was required for the conclusion. The authors both planned and performed fluorescense microscopy and therefore blinding of the data was not performed.

Reporting for specific materials, systems and methods

We require information from authors about some types of materials, experimental systems and methods used in many studies. Here, indicate whether each material, system or method listed is relevant to your study. If you are not sure if a list item applies to your research, read the appropriate section before selecting a response.

Materials & experimental systems

n/a	Involved in the study
☒	☐ Antibodies
☐	☒ Eukaryotic cell lines
☒	☐ Palaeontology and archaeology
☒	☐ Animals and other organisms
☒	☐ Human research participants
☒	☐ Clinical data
☒	☐ Dual use research of concern

Methods

n/a	Involved in the study
☒	☐ ChIP-seq
☒	☐ Flow cytometry
☒	☐ MRI-based neuroimaging

Eukaryotic cell lines

Policy information about cell lines

Cell line source(s)	- the SF21 cell line was obtained from the EMBL Protein Expression and Purification Core Facility (Heidelberg, Germany)<br>- the ESM243-1 and ESM243-2 yeast strains were generated in our lab by sporulation of the FY1679 yeast wild-type strain from Euroscarf (http://www.euroscarf.de/plasmid_details.php?accno=10000D)
Authentication	The ESM243-1 and ESM243-2 yeast strains were verified by marker analysis using standard yeast techniques. The SF21 cell line obtained from the EMBL Protein Expression and Purification Core Facility (Heidelberg, Germany) was not authenticated.
Mycoplasma contamination	Mycoplasma test was negative for all cell lines.
Commonly misidentified lines<br>(See ICLAC register)	No misidentified cell lines in this study.
